# Supplementary material for: Diagnostic delay of sarcoidosis: Protocol for an integrated systematic review
Source: PLoS One. 2023 Feb 22;18(2):e0269762. doi: 10.1371/journal.pone.0269762 (PMC9946231; doi:10.1371/journal.pone.0269762)
Supplement: S1 File — (DOCX) [file pone.0269762.s001.docx]

Diagnostic delay of Sarcoidosis: a protocol of an integrated systematic review

# Section 1: Administration Information

## Item 1. Title

Diagnostic delay of Sarcoidosis: a protocol of an integrated systematic review

## Item 2. Registration

This systematic review will be registered with PROSPERO.

## Item 3. Authors

Tergel Namsrai MD, MSc^1^

Anne Parkinson BA (Hons), AFHEA, PhD^1^

Dianne Gregory^1,4^

Elaine Kelly^1,4^

Christine Phillips MBBS, BMedSc, MA, MPH, DipEd, FRACGP, MD, AM^2^

Matthew Cook MBBS, PhD, FRACP, FRCPA, FFSc(RCPA)^3^

Jane Desborough RN, RM, MPH, PhD^1^*

### Item 3a. Affiliations

1. National Centre for Epidemiology and Population Health, Australian National University,

Canberra, Australia

2. Australian National University Medical School

3. John Curtin School of Medical Research, Australian National University

4. Sarcoidosis Australia

### *Correspondence

Jane Desborough, National Centre for Epidemiology and Population Health, Australian National University, 63, Eggleston Road, Acton ACT, 2601, Australia

Email: [Jane.Desborough@anu.edu.au](mailto:Jane.Desborough@anu.edu.au)

### Item 3b. Contributions

TN drafted the review protocol. All authors will contribute to the study. TN and AP are the primary reviewers. JD is the third reviewer and guarantor of the study.

## Item 4. Amendments

In the event of protocol amendments, date, explanation, and rationale of the amendment will be described in this section. The record will be in tabular format as shown below.

| Table1. Record of Amendments | | | | |
| --- | --- | --- | --- | --- |
| Date | Section | Original protocol | Revised protocol | Rationale |
| 09/12/2021 | Appendix 1. Search string | "sarcoidosis"[Title/Abstract] AND ("delay in diagnosis"[Title/Abstract] OR "diagnostic delay"[Title/Abstract] OR "misdiagnosis"[Title/Abstract] OR "time to diagnosis"[Title/Abstract] OR "incorrect diagnosis"[Title/Abstract] OR "missed diagnosis"[Title/Abstract] OR "delayed diagnosis"[Title/Abstract]) | "sarcoidosis"[Title/Abstract] AND ("delay in diagnosis"[Title/Abstract] OR "diagnostic delay"[Title/Abstract] OR "misdiagnosis"[Title/Abstract] OR "time to diagnosis"[Title/Abstract] OR "incorrect diagnosis"[Title/Abstract] OR "missed diagnosis"[Title/Abstract] OR "delayed diagnosis"[Title/Abstract]) OR “slow diagnosis”[Title/Abstract]) | After peer review “slow diagnosis” has been added to the search terms. The search string has been changed accordingly. |

## Item 5. Support

### Item 5a. Sources

This integrated systematic review is part of the “Missed opportunities in clinical practice: Tools to enhance healthcare providers’ awareness and diagnosis of rare diseases in Australia” project funded by the Commonwealth represented by Department of Health Australia (Grant ID 4-G5ZN0T7).

### Item 5b and 5c. Sponsor name and its role

The Commonwealth of Australia represented by the Department of Health has provided a grant for the “Missed opportunities in clinical practice: Tools to enhance healthcare providers’ awareness and diagnosis of rare diseases in Australia” project which includes this review.

# Section 2: Introduction

## Item 6. Rationale

Sarcoidosis is a systemic inflammatory granulomatous disease of unknown cause (Costabel and Hunninghake, 1999, James et al., 1976). Consequently, sarcoidosis can manifest in any organ including the lungs, skin, liver, joints, nervous system, and eyes (Grunewald et al., 2019, Ungprasert et al., 2019), but it most commonly affects the lungs, referred to as pulmonary sarcoidosis (PS) (Fernández-Ramón et al., 2021).

Sarcoidosis’ symptoms can be diverse ranging from acute epileptic seizures to subtle symptoms such as fatigue and pain syndromes that are subject to the organ involved (de Kleijn et al., 2009, Michielsen et al., 2006, Bakkers et al., 2009). Additionally, it lacks a single diagnostic test, and unified commonly used diagnostic criteria. Most importantly, sarcoidosis diagnosis relies on clinical manifestations along with radiological or histological evidence and exclusion of possible alternative diagnoses (Costabel and Hunninghake, 1999). The diagnosis is confirmed when both clinical and radiological findings are supported by histological evidence of non-necrotic granulomas on affected organs following biopsy.

Given the broad clinical presentations, lack of any diagnostic test or unified diagnostic criteria, and the exclusion approach of diagnosis, timely diagnosis of sarcoidosis is challenging. A recent study reported that it took 4.8 visits for a diagnosis of sarcoidosis to be reached and only 15% of people with Sarcoidosis were suspected of having Sarcoidosis in the first visit (Judson et al., 2003).

However, studies examining the overall diagnostic delay, contributing factors, and people’s experience of diagnostic delay in Sarcoidosis are scarce. Further studies on diagnostic delay are important for gaining clearer insight into diagnostic delays. This will inform future studies, interventions, tools, and health policies directed at enhancing diagnostic efficiency and patient experience of Sarcoidosis.

## Item 7. Objectives

The aim of this systematic review is to review the evidence regarding diagnostic delay in Sarcoidosis.

To this end, the systematic review will answer the following questions:

1. What are the causes and consequences of diagnostic delay of Sarcoidosis?
2. What evidence is there about patients’ experience of Sarcoidosis’ diagnostic delay?

# Section 3: Methods

## Item 8. Eligibility criteria

The studies will be selected according to the eligibility criteria developed using the PICOS tool (Methley et al., 2014)

Inclusion criteria:

- 1. Participants

We will include all studies examining people of all ages with all types of Sarcoidosis including pulmonary, cardiac, eye, kidney and gastrointestinal.

- 1. Exposure

We will include all studies examining delayed, incorrect diagnosis or missed diagnosis of Sarcoidosis (outlined above in section 1 Participants).

- 1. Comparison or control group

Given the aim of the study we will not include a control group.

- 1. Outcome of interest

The main outcomes of interest are time to diagnosis, factors associated with diagnostic delay and patients’ experiences of diagnosis of Sarcoidosis.

We will include quantitative studies with adequately reported data (the actual words of the participant or the field notes of observers) as well as findings (the results of the researcher’s analysis and interpretation).

We will include qualitative studies with patients’ experience of diagnostic delay for Sarcoidosis.

- 1. Timing

There will be no restriction in timing of the studies.

- 1. Setting

There will be no restriction in settings.

- 1. Study design

We will include all types of study design such as observational studies, clinical trials, case-reports, and qualitative studies, except for review articles. However, the reference lists of review articles will be hand searched for relevant papers.

- 1. Language

We will include studies published in English, Indonesian and German.

## Item 9. Information sources

Electronic database and grey literature searches will be conducted.

1. Electronic database searches: PUBMED/MEDLINE, Scopus, and ProQuest.
2. Other methods to identify relevant literature: grey literature will be searched using Google Scholar.

The search strategy will be developed using the PICOS method as recommended in the Cochrane systematic review handbook (Methley et al., 2014).

## Item 10. Search strategy

The search strategy was developed to ensure reproducibility and increase transparency following the PRISMA-P checklist (Moher et al., 2015). Research questions and search terms were developed using the PICOS tool (Population/Intervention/Comparison/Outcomes/Study Design) to enhance the scientific literature by ensuring reliability and homogeneity of search results (Methley et al., 2014).

The primary source of literature will be a systematic search of multiple electronic databases (from inception onwards): PubMed/Medline, Scopus, and ProQuest. Sources of grey literature will also be searched. Grey literature search will be conducted through Open Access Theses and Dissertation (<https://oatd.org/> ), ProQuest thesis and dissertations and The National Library of Australia. Additionally, reference lists of selected studies and review articles will be searched. All settings and study design will be considered.

Search terms was developed in collaboration with research team members and peer reviewed (TN, AP, JD, MC, CP) using the PRESS checklist (McGowan et al., 2016). Search terms was combined using Boolean operators “AND” and “OR”. Preliminary exploratory searches of the literature were undertaken (15 October 2021) to inform the final search strategy and determine outcomes. The final search strategy that was developed and used on PUBMED/MEDLINE database is shown in Appendix 1.

## Item 11. Study records

### Item 11a. Data management

The literature search results will be imported to Covidence, an internet-based software that facilitates collaboration between reviewers and ensures independent review of the literature (Veritas Health Innovation)

### Item 11b. Selection process

Two review authors will independently screen the titles and abstract of literature search against the pre-developed inclusion criteria (TN and AP). Any conflict in the title and abstract screening process will be discussed among the review team and will be resolved by a third reviewer (JD). Full reports for all studies that meet the inclusion criteria or where there is any uncertainty will be obtained. Review authors will then screen full text reports according to the inclusion criteria. The reasons for excluding studies will be recorded. Authors will not be blinded to the study types, journals, and authors during this process.

### Item 11c. Data collection

After the study selection process is complete, a data extraction tool will be designed, peer reviewed and piloted. In the piloting process, two independent reviewers will extract data independently and in duplicate from 5 studies each and compare their results to establish agreement and validity of the data extraction tool. Any disagreements will be resolved through discussion and conflicts resolved by a third reviewer (JD). We will contact study authors to resolve any uncertainties about extracted data.

## Item 12. Data items

The following data items will be extracted:

Identification of the study

Journal,

Authors,

Year,

Citation,

Research center/university/hospital/organization

Conflict of interest,

Funding/sponsorship.

Methods

Study aim,

Study design,

Participant demographics,

Recruitment process,

Inclusion,

Exclusion criteria,

Statistical analysis.

Main findings

Exposure details,

Diagnostic delays,

Factors associated with diagnostic delay,

Patients’ experience,

Relevant outcomes.

## Item 13. Outcomes and prioritization

Primary outcome

Diagnostic delay time (time from symptom onset to correct diagnosis) in people living with Myositis

Secondary outcomes

Patient’s experiences related to diagnostic delay

Causes and consequences of diagnostic delay

## Item 14. Quality assessment or risk of bias

The selected studies will be assessed for methodological quality or risk of bias using the Mixed Methods Appraisal Tool (MMAT) designed to critically appraise mixed method studies included in systematic reviews (Hong et al., 2018). Two independent review authors will conduct the quality appraisal. Any conflicts will be resolved with discussion and a third reviewer’s vote (JD).

## Item 15. Data synthesis

A systematic narrative synthesis will be undertaken to explore the findings of included studies in relation to time from symptom onset to diagnosis, and people’s experiences related to delayed diagnosis in line with guidance from the Centre for Reviews and Dissemination (Centre for Reviews and Dissemination., 2009).

If extracted quantitative data are homogenous, a meta-analysis will be conducted using a random-effects model. Extracted qualitative data will be meta-synthesized using meta-aggregation. In line with meta-aggregation methods, findings (processed data) from qualitative studies will be extracted and aggregated into a single set of categories, which will then be further aggregated and synthesised into a set of statements that are meaningful for clinical practice.

Further methods and stages of meta-analysis will be discussed if collected data is quantitively synthesizable. The findings from the quantitative and qualitative studies will be reported separately; however, the discussion will be integrative of both.

## Item 16. Confidence in cumulative estimate

If a meta-analysis is conducted, the quality/certainty of evidence for all quantitative outcomes will be judged using the Grading of Recommendations Assessment, Development and Evaluation (GRADE) working group methodology (Balshem et al., 2011). Certainty of the body of evidence will be assessed across domains of risk of bias, consistency of effect, imprecision, indirectness, and publication bias. The certainty will be reported in four levels: high, moderate, and very low.

## Item 17. Timeline and stages of review

| Table 2. Timeline and process of systematic review | | | |
| --- | --- | --- | --- |
|  | Started | Completed | Timeline |
| Protocol development | Yes | Yes | November, 2021 |
| Search strategy development | Yes | Yes | December 2021 |
| Preliminary literature search | Yes | Yes | October, 2021 |
| Literature search | Yes | No | December, 2021 |
| Piloting of the study selection process | No | No | April, 2022 |
| Study selection | No | No | April-May, 2022 |
| Quality appraisal | No | No | June, 2022 |
| Data extraction | No | No | June-July 2022 |
| Data synthesis | No | No | July-August, 2022 |
| Writing paper | No | No | August-September, 2022 |

## Version history

| Table 3. Version history of systematic review protocol | | |
| --- | --- | --- |
| Date | Version number | Explanation |
| 09 December 2021 | Version 1.0 | First draft of review, “Diagnostic delay of Sarcoidosis: a protocol of an integrated systematic review” |
| 16 December 2021 | Version 2.0 | Updated version of review, “Diagnostic delay of Sarcoidosis: a protocol of an integrated systematic review” |

# Appendix 1.

Search terms used to develop final search string for PubMed search conducted on 15^th^ of October 2021.

## Search string:

"sarcoidosis"[Title/Abstract] AND ("delay in diagnosis"[Title/Abstract] OR "diagnostic delay"[Title/Abstract] OR "misdiagnosis"[Title/Abstract] OR "time to diagnosis"[Title/Abstract] OR "incorrect diagnosis"[Title/Abstract] OR "missed diagnosis"[Title/Abstract] OR "delayed diagnosis"[Title/Abstract] OR “slow diagnosis” [Title/Abstract])

## Search history

| Table 4. Search history of Myositis search for PUBMED/MEDLINE | | | | |
| --- | --- | --- | --- | --- |
| Search number | Query | | Search Details | Results |
| 11 | #1 AND #10 | | "sarcoidosis"[Title/Abstract] AND ("delay in diagnosis"[Title/Abstract] OR "diagnostic delay"[Title/Abstract] OR "misdiagnosis"[Title/Abstract] OR "time to diagnosis"[Title/Abstract] OR "incorrect diagnosis"[Title/Abstract] OR "missed diagnosis"[Title/Abstract] OR "delayed diagnosis"[Title/Abstract] OR "slow diagnosis"[Title/Abstract]) | 164 |
| 10 | #2 OR #3 OR #4 OR #5 OR #6 OR #7 OR #8 OR #9 | | "delay in diagnosis"[Title/Abstract] OR "diagnostic delay"[Title/Abstract] OR "misdiagnosis"[Title/Abstract] OR "time to diagnosis"[Title/Abstract] OR "incorrect diagnosis"[Title/Abstract] OR "missed diagnosis"[Title/Abstract] OR "delayed diagnosis"[Title/Abstract] | 38,186 |
| 9 | "slow diagnosis"[Title/Abstract] | | "slow diagnosis"[Title/Abstract] | 8 |
| 8 | "delayed diagnosis"[Title/Abstract] | "delayed diagnosis"[Title/Abstract] | | 8,589 |
| 7 | "missed diagnosis"[Title/Abstract] | "missed diagnosis"[Title/Abstract] | | 2,399 |
| 6 | "incorrect diagnosis"[Title/Abstract] | "incorrect diagnosis"[Title/Abstract] | | 1,299 |
| 5 | "time to diagnosis"[Title/Abstract] | "time to diagnosis"[Title/Abstract] | | 2,661 |
| 4 | "misdiagnosis"[Title/Abstract] | "misdiagnosis"[Title/Abstract] | | 16,610 |
| 3 | "diagnostic delay"[Title/Abstract] | "diagnostic delay"[Title/Abstract] | | 3,203 |
| 2 | "delay in diagnosis"[Title/Abstract] | "delay in diagnosis"[Title/Abstract] | | 5,967 |

# Reference

BAKKERS, M., MERKIES, I. S., LAURIA, G., DEVIGILI, G., PENZA, P., LOMBARDI, R., HERMANS, M. C., VAN NES, S. I., DE BAETS, M. & FABER, C. G. 2009. Intraepidermal nerve fiber density and its application in sarcoidosis. *Neurology,* 73**,** 1142-8.

BALSHEM, H., HELFAND, M., SCHÜNEMANN, H. J., OXMAN, A. D., KUNZ, R., BROZEK, J., VIST, G. E., FALCK-YTTER, Y., MEERPOHL, J., NORRIS, S. & GUYATT, G. H. 2011. GRADE guidelines: 3. Rating the quality of evidence. *J Clin Epidemiol,* 64**,** 401-6.

CENTRE FOR REVIEWS AND DISSEMINATION. 2009. *Systematic Reviews,* University of York, York Publishing Services Ltd.

COSTABEL, U. & HUNNINGHAKE, G. 1999. ATS/ERS/WASOG statement on sarcoidosis. Sarcoidosis Statement Committee. American Thoracic Society. European Respiratory Society. World Association for Sarcoidosis and Other Granulomatous Disorders. *European Respiratory Journal,* 14**,** 735-737.

DE KLEIJN, W. P., DE VRIES, J., LOWER, E. E., ELFFERICH, M. D., BAUGHMAN, R. P. & DRENT, M. 2009. Fatigue in sarcoidosis: a systematic review. *Current Opinion in Pulmonary Medicine,* 15**,** 499-506.

FERNÁNDEZ-RAMÓN, R., GAITÁN-VALDIZÁN, J. J., SÁNCHEZ-BILBAO, L., MARTÍN-VARILLAS, J. L., MARTÍNEZ-LÓPEZ, D., DEMETRIO-PABLO, R., GONZÁLEZ-VELA, M. C., CIFRIÁN, J., CASTAÑEDA, S., LLORCA, J., GONZÁLEZ-GAY, M. A. & BLANCO, R. 2021. Epidemiology of sarcoidosis in northern Spain, 1999-2019: A population-based study. *European Journal of Internal Medicine,* 91**,** 63-69.

GRUNEWALD, J., GRUTTERS, J. C., ARKEMA, E. V., SAKETKOO, L. A., MOLLER, D. R. & MULLER-QUERNHEIM, J. 2019. Sarcoidosis. *Nat Rev Dis Primers,* 5**,** 45.

HONG, Q. N., FÀBREGUES, S., BARTLETT, G., BOARDMAN, F., CARGO, M., DAGENAIS, P., GAGNON, M.-P., GRIFFITHS, F., NICOLAU, B., O’CATHAIN, A., ROUSSEAU, M.-C., VEDEL, I. & PLUYE, P. 2018. The Mixed Methods Appraisal Tool (MMAT) version 2018 for information professionals and researchers. *Education for Information,* 34**,** 285-291.

JAMES, D. G., TURIAF, J., HOSODA, Y., WILLIAMS, W. J., ISRAEL, H. L., DOUGLAS, A. C., SILTZBACH, L. E. & MEMBERS, P. 1976. DESCRIPTION OF SARCOIDOSIS: REPORT OF THE SUBCOMMITTEE ON CLASSIFICATION AND DEFINITION. *Annals of the New York Academy of Sciences,* 278**,** 742-742.

JUDSON, M. A., THOMPSON, B. W., RABIN, D. L., STEIMEL, J., KNATTEREUD, G. L., LACKLAND, D. T., ROSE, C., RAND, C. S., BAUGHMAN, R. P. & TEIRSTEIN, A. S. 2003. The Diagnostic Pathway to Sarcoidosis*. *Chest,* 123**,** 406-412.

MCGOWAN, J., SAMPSON, M., SALZWEDEL, D. M., COGO, E., FOERSTER, V. & LEFEBVRE, C. 2016. PRESS Peer Review of Electronic Search Strategies: 2015 Guideline Statement. *Journal of Clinical Epidemiology,* 75**,** 40-46.

METHLEY, A. M., CAMPBELL, S., CHEW-GRAHAM, C., MCNALLY, R. & CHERAGHI-SOHI, S. 2014. PICO, PICOS and SPIDER: a comparison study of specificity and sensitivity in three search tools for qualitative systematic reviews. *BMC Health Services Research,* 14**,** 579.

MICHIELSEN, H. J., DRENT, M., PEROS-GOLUBICIC, T. & DE VRIES, J. 2006. Fatigue Is Associated With Quality of Life in Sarcoidosis Patients*. *Chest,* 130**,** 989-94.

MOHER, D., SHAMSEER, L., CLARKE, M., GHERSI, D., LIBERATI, A., PETTICREW, M., SHEKELLE, P., STEWART, L. A. & GROUP, P.-P. 2015. Preferred reporting items for systematic review and meta-analysis protocols (PRISMA-P) 2015 statement. *Systematic Reviews,* 4**,** 1.

UNGPRASERT, P., RYU, J. H. & MATTESON, E. L. 2019. Clinical Manifestations, Diagnosis, and Treatment of Sarcoidosis. *Mayo Clinic Proceedings: Innovations, Quality & Outcomes,* 3**,** 358-375.

VERITAS HEALTH INNOVATION. *Covidence systematic review software* [Online]. Melbourne, Australia. Available: Available at www.covidence.org [Accessed].
