# Supplementary material for: Diagnostic delay of sarcoidosis: Protocol for an integrated systematic review
Source: PLoS One. 2023 Feb 22;18(2):e0269762. doi: 10.1371/journal.pone.0269762 (PMC9946231; doi:10.1371/journal.pone.0269762)
Supplement: Attachment — Submitted filename: Response to reviewers.docx [file pone.0269762.s002.docx]

| Table 1. Response to reviewer’s | | | |
| --- | --- | --- | --- |
|  | Comments | Action taken/Response to the reviewer | Edited manuscript |
| Review 1’s comments | |  |  |
| 1. | Thank you for asking me to review this protocol. The intention is laudable in that the diagnosis of sarcoidosis is often delayed, in part due to the fact that it is uncommon, but also because the diseases manifests in multiple ways and in different organs.  The systematic review does not indicate how many articles they expect to identify nor are there many in the literature to date. This reviewer finds the publication of protocols of this nature somewhat unhelpful and would prefer to see the final article as publishes in the Cochrane database or elsewhere, rather than piecemeal. | Thank you for your comment. Inclusion of expected results would be helpful. Indeed, we are hoping to publish the results of this review when finished.  Preliminary search was conducted on PUBMED during the development of search string. As suggested, we have added results column to Table 1 that shows the preliminary searches and a clarification on the preliminary search in the manuscript. | On page 8 line 120:  *“The preliminary search conducted during the development of the search string on PUBMED/MEDLINE identified 162 relevant titles (Table 1).”* |
| 2 | The main issues are:  The appears to be the same protocol already available on the internet, raising the issue of duplicate publication. https://www.medrxiv.org/content/10.1101/2022.05.30.22275771v1 https://www.crd.york.ac.uk/PROSPEROFILES/307236_PROTOCOL_20220127.pdf | Thank you for highlighting an important issue. Both of the protocols mentioned are copies of our systematic review’s protocol.  The first link (<https://www.medrxiv.org/content/10.1101/2022.05.30.22275771v1> ) is a copy of a preprint for the manuscript that was submitted to Plos One.  The second link (<https://www.crd.york.ac.uk/PROSPEROFILES/307236_PROTOCOL_20220127.pdf>) is a copy of our protocol that was uploaded to PROSPERO application for registration. This original protocol that was developed according to the PROSPERO template was attached in the Supplementary file.  As suggested, changes were made to include our preprint and protocol on PROSPERO registration in the manuscript | On page 14 line 196:  ***“Protocol versions***  *Two copies of the current protocol are available on PROSPERO registration and medRxiv(1). ”* |
| 3 | Is this intended to be a Cochrane review? If so, it should be listed as such and the title on their website. | No, this is not intended to be a Cochrane review. |  |
| 4 | Specific comments:  The choice of languages for the available articles is curious, but presumably reflects the skills of the authors- it would be useful to include other languages particularly Japanese which has a large body of literature on the topic of sarcoidosis. | We are interested to know that there are large body of literature in Japanese. Unfortunately, none of the members of the research team or colleagues in our department read/speaks Japanese. |  |
| 5 | The introduction is somewhat lacking in balance: it is not a rare disease (10-40 in 100,000). Seizures not are a common presentation- ophthalmological, respiratory and rheumatological symptoms are much more common. | Thank you for pointing out an important issue.  We described sarcoidosis as a rare disease based on the Australian Department of Health and Aged Care’s description of rare diseases. To aid in the clarification, we have added reference of the information in the introduction.  We aimed to show how complex and rare clinical presentation of sarcoidosis can be along with common features. However, this seems not to be the case. Therefore, as suggested the introduction part was altered in a way that provides more balance between common and rare clinical features of sarcoidosis. | On page 6 line 76:  *“Sarcoidosis is a rare systemic inflammatory granulomatous disease of unknown cause with broad manifestation ranging from more common signs (cough, shortness of breath and eye signs) to rare presentations (acute epileptic seizures) and subtle symptoms such as fatigue and pain that are subject to the organ involved (Michielsen, Peros-Golubicic et al. 2007, Bakkers, Merkies et al. 2009, de Kleijn, De Vries et al. 2009, Department of Health and Aged Care 2022).”* |
| Reviewer 2’s comments | |  |  |
| 1 | This is expected to be a good study at conception with valid research questions and expected outcomes.  Authors have expressed their concerns on the limited number of available studies that deal with diagnostic delay in sarcoidosis. Has there been a preliminary literature search that can assure authors of being able to get reasonable number of manuscripts for review? | Thank you for highlighting an important issue. Preliminary search was conducted on PUBMED during the development of search string which identified 162 relevant titles. After discussion with research team, we determined this as a reasonable amount of titles. To clarify this, we have included the results of the preliminary search in the manuscript and Table 1. | On page 8 line 120:  *“The preliminary search conducted during the development of the search string on PUBMED/MEDLINE identified 162 relevant titles (Table 1).”* |
| 2 | How do the authors plan to do statistical analysis, have they got the skill or this will be sourced out? | This is an important point. Thank you for raising this question.  The data analysis process will be conducted on R by our research team who has relevant knowledge and experience of R programming as well as health research. Additionally, we have the support of statistical expertise in the National Centre for Epidemiology and Health at the Australian National University whom we will consult if required.  The process of data analysis has been added to the manuscript as suggested. | On page 13 line 180:  *“Meta-analysis including pooling of diagnostic delay and subgroup analysis will be conducted using R studio version 4.2.1 using “meta” package.”* |
| 3 | Assuming there are enough manuscript from literature search, I believe this study will be helpful to both patients as well as clinicians. recommendations will be helpful in looking at causes and consequences of delay in diagnosis and way forward. | Thank you for reviewing our protocol and providing comments. We hope to provide insight into diagnostic delay in sarcoidosis and further facilitate in enhancing diagnostic efficiency in sarcoidosis. |  |
