# Supplementary material for: Diagnostic delay of sarcoidosis: Protocol for an integrated systematic review
Source: PLoS One. 2023 Feb 22;18(2):e0269762. doi: 10.1371/journal.pone.0269762 (PMC9946231; doi:10.1371/journal.pone.0269762)
Supplement: Attachment — Submitted filename: Response to reviewers_final_submission.docx [file pone.0269762.s003.docx]

| Table 1. Response to reviewers on the manuscript titled: Diagnostic delay of sarcoidosis: protocol for an integrated systematic review | | |
| --- | --- | --- |
| Comments | Response to the reviewer | Edits in the manuscript |
| Editorial comments | | |
| Comment 1  Please specify your study and mentioned details eligibility criteria. | Thank you for your comment. A separate section on study selection (inclusion and exclusion criteria) has been added to provide more detail on the eligibility of the studies identified through the systematic search of the literature. | **Study selection (inclusion and exclusion criteria)**  The eligibility of the studies identified through the literature search will be determined according to the pre-developed PICOS eligibility criteria outlined in Table 2.  **Inclusion criteria**  *Population*  Studies examining people with all types of sarcoidosis and of all ages.  *Intervention/Exposure*  Studies about delayed diagnosis, incorrect diagnosis, missed diagnosis or slow diagnosis of sarcoidosis.  *Comparison*  Given the nature of the study there will be no comparison group.  *Outcomes*  Studies that have measures on diagnostic delay, factors of diagnostic delay, and people with sarcoidosis’ experiences of diagnostic delay.  *Study design*  Qualitative, quantitative and mixed methods will be included if they meet the PICOS eligibility criteria.  *Other*  No setting or publication date limitations will be applied. Only studies in English, German and Indonesian will be included.  **Exclusion criteria**  Studies on animals, review articles, clinical trials, randomized trials not reporting on delayed diagnosis, incorrect diagnosis, missed diagnosis or slow diagnosis of sarcoidosis. |
| Comment 2  Please provide screening methodology, selection methodology | Separate sections on study selection and the study screening process have been added as suggested. | **Study selection (inclusion and exclusion criteria) –** please see changes as described above in response to comment 1  **Screening**  Studies identified through the systematic search will undergo title and abstract screening followed by full text screening for those remaining.  Two review authors (** and **) will independently screen titles and abstracts, and full texts against the pre-developed inclusion criteria. Any conflicts will be discussed and resolved by consensus with a third reviewer (**). Exclusion rationales will be recorded. |
| Comment 3  Please provide exclusion criteria with details | Thank you for highlighting an important factor. A detailed exclusion criteria section has been added to the study selection and Table 2 as well. | **Exclusion criteria**  Studies on animals, review articles, clinical trials, randomized trials not reporting on delayed diagnosis, incorrect diagnosis, missed diagnosis or slow diagnosis of sarcoidosis. |
| Comment 4  Please provide possible limitation in the study. | A separate strengths and limitations section has been added to the manuscript. | **Strengths and limitations**  The main strength of this systematic review will be the inclusion of all study types (qualitative, quantitative, and mixed methods) to integrate and analyse the current literature about diagnostic delay, its determinants and consequences, including people’s experiences of delayed diagnosis in all types of sarcoidosis. A potential limitation of the study will be insufficient studies with data on ethnic or gender differences in diagnostic delay. While there will be sufficient studies exploring pulmonary sarcoidosis, there may be insufficient studies for some rarer types to identify factors associated with delays in diagnosing pulmonary sarcoidosis. |
| Comment 5  The current information given were incomplete. Please provide background of study, problem statement and rational of the study. | We have expanded the introduction to include a background to the study and added a problem statement as requested. The rationale of the study has also been amended as suggested. | **Background**  Sarcoidosis is a rare systemic inflammatory granulomatous disease. The incidence and prevalence of sarcoidosis vary across countries, and by ethnicity and gender. In studies using national datasets or large cohorts, the reported incidence is highest in northern Europe at 11.5 per 100,000 per year in Sweden (1) and 11.3-14.8 per 100,000 per year in Denmark (2). *Lower incidences have been reported in Asian countries (3-5). Intra-country differences attributed to race are reported in the USA where the incidence among African Americans is higher than that of other Americans (6). In Canada, migrants accounted for 10% of cases, with South East Asian migrants disproportionately represented (7). Higher incidence rates among women have been reported in some studies, (6, 8, 9) but not in others (10) The pattern of disease may differ for women, and they may be diagnosed at a later age (1, 11) (12).*  Sarcoidosis is a disease of unknown cause, which can manifest in any organ including heart, skin, liver, joints, nervous system and eyes (13, 14), but it most commonly affects the lungs (9). Its symptoms reflect the range of organs involved, from symptoms attributable to its more common pulmonary manifestation to unusual presentations involving other organs and subtle symptoms such as fatigue and pain (15-18). *Sarcoidosis can be asymptomatic, and be discovered incidentally; in one study, 13.6% of cases of pulmonary sarcoidosis were asymptomatic (19).* There is no single diagnostic test for sarcoidosis, nor a unified, commonly used set of diagnostic criteria. Diagnosis of sarcoidosis relies on clinical manifestations along with radiological or histological evidence and exclusion of possible alternative diagnoses (20).  *The reported delay of onset of sarcoidosis ranges from six months to nine years depending on the organ involvement (21, 22). In one study in the USA, only 15.3% of cases were diagnosed at the first visit (23). Delays in diagnosis of sarcoidosis can lead to extension of the disease, organ impairment and can be attended by physical suffering and exhaustion among patients.*  *While there are studies investigating determinants of delay, there have been few systematic reviews of these studies, and none incorporating qualitative and quantitative research to incorporate people’s experiences of the processes associated with diagnostic delay.* We aim to systematically review the evidence about diagnostic delay in sarcoidosis to elucidate the factors associated with diagnostic delay for this disease in different contexts and settings, and the consequences of this delay for people with sarcoidosis. *More detailed information about the factors associated with delay may help throw light on points of intervention, and strategies to ensure earlier diagnosis.* |
| Comment 6  Please provide statistical signification of the sarcoidosis disease? such as prevalence rate all around the world. | Apologies for not including this important information. We have added information about incidence rates as the study focuses on the diagnostic delay, Incidence rate is drawn from multiple countries around the world, it has been added as a range due to the limited global studies on the prevalence of sarcoidosis. | Sarcoidosis is a rare systemic inflammatory granulomatous disease. The incidence and prevalence of sarcoidosis vary across countries, and by ethnicity and gender. *In studies using national datasets or large cohorts, the reported incidence is highest in northern Europe at 11.5 per 100,000 per year in Sweden and 11.3-14.8 per 100,000 per year in Denmark (2). Lower incidences have been reported in Asian countries (3-5). Intra-country differences attributed to race are reported in the USA where the incidence among African Americans is higher than that of other Americans (6). In Canada, migrants accounted for 10% of cases, with South East Asian migrants disproportionately represented (7). Higher incidence rates among women have been reported in some studies, (6, 8, 9) but not in others (10) The pattern of disease may differ for women, and they may be diagnosed at a later age (1, 11) (12).* |
| Comment 7  Please provide data management tools. | Thank you for your comment. A separate section on data management added. | **Data management**  To ensure an independent review and screening process of the studies identified through the literature search we will use an internet-based software, Covidence, that facilitates collaboration between reviewers while maintaining independence (24). All search results will be imported to Covidence, and research team members will screen individually while being blinded to the choices made by other members. Any conflicts will be resolved through discussion and consensus following each screening process. |
| Comment 8  Please provide data collection process in detail | A detailed data extraction/collection section has been added as requested. | **Data extraction/ Data collection**  Following completion of the study selection and screening, data extraction will be conducted in four stages: 1) development of a data extraction tool, 2) peer-review of the data extraction tool, 3) piloting of the data extraction tool, and 4) final data extraction. The data extraction tool will be designed by ** based on discussions with the research team and then reviewed by the team. This will be followed by a piloting stage where two reviewers (** and **) will independently extract data from the same five studies and compare their results to establish consensus and validity of the data extraction tool. Final data extraction will be conducted by an individual reviewer. |
| Comment 9  Please provide data items, data synthesis methodology. All information must written in detail under separate heading. | Thank you for pointing out this important issue. A separate and detailed section on data items and data synthesis has been added. | **Data items**  The following data items will be extracted from the included studies:   1. Identification of the study   This will include name of the journal, authors, publication year, short citation, research center/university/hospital/organisation, conflict of interest, and funding/sponsorship.   2. Methods   This will include study aim, study design, diagnostic criteria used, participant demographics (mean age, sex, number of participants, ethnicity, country), recruitment process, inclusion, exclusion criteria, and statistical analysis.   3. Main findings   This will include diagnosis (sarcoidosis location), type of diagnostic delay (doctor’s delay or patient’s delay), diagnostic delay in months (standard deviation, standard error, confidence interval of the diagnostic delay) factors of delay (estimates, odds ratio, relative risk, standard deviations, standard errors and confidence intervals), main symptoms, patients’ experiences, and other relevant outcomes.  **Data synthesis**  The extracted data items will be exported to Excel (spreadsheet software) (20). The data synthesis will be conducted using R programming running under R studio version 4.2.1. The data synthesis process will comprise four stages: 1) data checking and cleaning, 2) data conversion, 3) descriptive statistics, 4) data examination for eligibility for inclusion in meta-analysis. In the data checking and cleaning stage, all extracted data items will be checked for misspelling and missing data will be double checked to ensure any data is not mistakenly left out. This will be followed by the conversion of outcomes to unified units (i.e., delay in diagnosis will be converted to months). In this stage, all data items will be labelled (i.e., delay in diagnosis will be labelled as a numerical value). This will be followed by summarising descriptive statistics of the included studies which will be used to examine the possibility of conducting a meta-analysis. |
| Comment 10  Please give PICO in detail under each heading. | A detailed section with separate headings for PICOS has been added as requested. | **Study selection (inclusion and exclusion criteria)**  The eligibility of the studies identified through the literature search will be determined according to the pre-developed PICOS eligibility criteria outlined in Table 2.  **Inclusion criteria**  *Population*  Studies examining people with all types of sarcoidosis and of all ages.  *Intervention/Exposure*  Studies about delayed diagnosis, incorrect diagnosis, missed diagnosis or slow diagnosis of sarcoidosis.  *Comparison*  Given the nature of the study there will be no comparison group.  *Outcomes*  Studies that have measures on diagnostic delay, factors of diagnostic delay, and people with sarcoidosis’ experiences of diagnostic delay.  *Study design*  Qualitative, quantitative and mixed methods will be included if they meet the PICOS eligibility criteria.  *Other*  No setting or publication date limitations will be applied. Only studies in English, German and Indonesian will be included. |
| Comment 11  Please provide implication of study protocol and what will be benefit of this protocol. | Thank you for your comment. Implications of the study protocol have been added. | **Discussion**  In this systematic review protocol, a detailed plan of all steps of the review including protocol development, search strategy, study selection, data extraction, quality assessment, and data synthesis has been described to ensure the production of unbiased evidence regarding the diagnostic delay of sarcoidosis, its factors, and the experiences of diagnostic delay among people with sarcoidosis. Knowledge gained from this review may throw light on ways to improve delays in diagnosis across different subpopulations, and with different disease presentations. |
| Comment 12  Include some more tool to assess and compare bias in different studies. The current methodology is insufficient. | Thank you for pointing out an important issue. We have included the MMAT as the methodological evaluation and NOS as an alternative choice if the included studies are all quantitative. We have also included an assessment of risk of bias as suggested. | **Assessment of risk of bias**  The risk of bias of the included studies will be assessed through examining data presented as a funnel plot, a scatter plot of the effect sizes against the study sample (27). Visual inspection of asymmetry of the funnel plot will initially be conducted, with consideration of causes, including heterogeneity, reporting bias, publication bias and chance. In cases of funnel plot asymmetry, when there are more than ten studies included in the meta-analysis, further tests for funnel plot asymmetry will be used to assess the cause of bias (28). |
| Reviewer #1 | | |
| Comment 1  The authors have responded to the queries raised. This reviewer would prefer to see the main findings rather than just the protocol. | Thank you for reviewing the manuscript. The results of the systematic review will be published when completed along with the data. |  |
| Reviewer #2 | | |
| Comment 1  Previous comments have been sufficiently addressed. The outcome of this study is promising and will most likely fulfil objectives of the study and contributes to the body of knowledge in this field. | Thank you for reviewing the manuscript and for your supportive comments. The results of this review will be published when completed and we hope this will contribute to the body of knowledge in diagnosis of sarcoidosis. |  |
| Reviewer #3 | | |
| Comment 1 Lines 132-135: How will the authors examine patients’ experiences associated with diagnostic delays? This is not described in detail. Will there be a survey? Will evidence be based on published literature or follow up with respective authors? In the event that there are not enough studies detailing patients’ experiences, how do the authors plan to make up for that? | Thank you for your comment.  In this review we will review the available evidence of people’s experience of diagnostic delay of sarcoidosis. This evidence would most often be reported through the use of surveys or qualitative studies, including individual interviews or focus groups with people living with sarcoidosis. For this reason, we will include all types of studies (quantitative, qualitative and mixed methods).  If there is a lack of studies on people’s experience in diagnostic delay of sarcoidosis, due to the guidelines, we cannot take any additional steps as it may introduce bias to the study. However, in that case, it would mean we have identified a gap in the literature and future studies could focus on closing that gap and bringing more knowledge on the topic. |  |
| Comment 2  Since there are no limitations on publication dates, it would be interesting to see if the study shows a trend of stagnancy or improvement in diagnostic delay over the years. | Thank you for the interesting point. Yes, looking at the trend would bring in good idea of the diagnostic improvement over the years. We can do an analysis on the year of publication. This was reflected in the manuscript as well. | The main analysis will be pooled diagnostic delay in each type of sarcoidosis in conjunction with subgroup analyses (i.e., types of sarcoidosis, health service utilization, year of publication). |
| Comment 3  I totally agree with reviewer 1 about publishing the protocol as part of a complete systematic review and meta-analysis manuscript because that will be more informative and helpful. | Thank you for your comment. We agree publishing the results will be important when the review is complete.  However, we are keen to publish this protocol as a methodological paper and as a foundation for our research and as a guide for future researchers. |  |

1.	Arkema EV, Grunewald J, Kullberg S, Eklund A, Askling J. Sarcoidosis incidence and prevalence: a nationwide register-based assessment in Sweden. European Respiratory Journal. 2016;48(6):1690-9.

2.	Sikjær MG, Hilberg O, Ibsen R, Løkke A. Sarcoidosis: A nationwide registry-based study of incidence, prevalence and diagnostic work-up. Respiratory Medicine. 2021;187:106548.

3.	Morimoto T, Azuma A, Abe S, Usuki J, Kudoh S, Sugisaki K, et al. Epidemiology of sarcoidosis in Japan. European Respiratory Journal. 2008;31(2):372-9.

4.	Yoon H-Y, Kim HM, Kim Y-J, Song JW. Prevalence and incidence of sarcoidosis in Korea: a nationwide population-based study. Respiratory Research. 2018;19(1):158.

5.	Park JE, Kim YS, Kang MJ, Kim CJ, Han CH, Lee SM, et al. Prevalence, incidence, and mortality of sarcoidosis in Korea, 2003–2015: A nationwide population-based study. Respiratory Medicine. 2018;144:S28-S34.

6.	Baughman RP, Field S, Costabel U, Crystal RG, Culver DA, Drent M, et al. Sarcoidosis in America. Analysis Based on Health Care Use. Ann Am Thorac Soc. 2016;13(8):1244-52.

7.	Fidler LM, Balter M, Fisher JH, To T, Stanbrook MB, Gershon A. Epidemiology and health outcomes of sarcoidosis in a universal healthcare population: a cohort study. European Respiratory Journal. 2019;54(4):1900444.

8.	Akram MJ, Khalid U, Abu Bakar M, Butt FM, Ashraf MB. Sarcoidosis: epidemiology, characteristics, and outcomes over 10 years - a single-center study in Pakistan. Expert Review of Respiratory Medicine. 2021:1-11.

9.	Fernández-Ramón R, Gaitán-Valdizán JJ, Sánchez-Bilbao L, Martín-Varillas JL, Martínez-López D, Demetrio-Pablo R, et al. Epidemiology of sarcoidosis in northern Spain, 1999-2019: A population-based study. European Journal of Internal Medicine. 2021;91:63-9.

10.	Ungprasert P, Crowson CS, Matteson EL. Influence of Gender on Epidemiology and Clinical Manifestations of Sarcoidosis: A Population-Based Retrospective Cohort Study 1976-2013. Lung. 2017;195(1):87-91.

11.	Brito-Zerón P, Sellarés J, Bosch X, Hernández F, Kostov B, Sisó-Almirall A, et al. Epidemiologic patterns of disease expression in sarcoidosis: age, gender and ethnicity-related differences. Clin Exp Rheumatol. 2016;34(3):380-8.

12.	Judson MA, Boan AD, Lackland DT. The clinical course of sarcoidosis: presentation, diagnosis, and treatment in a large white and black cohort in the United States. Sarcoidosis Vasc Diffuse Lung Dis. 2012;29(2):119-27.

13.	Grunewald J, Grutters JC, Arkema EV, Saketkoo LA, Moller DR, Muller-Quernheim J. Sarcoidosis. Nat Rev Dis Primers. 2019;5(1):45.

14.	Ungprasert P, Ryu JH, Matteson EL. Clinical Manifestations, Diagnosis, and Treatment of Sarcoidosis. Mayo Clinic Proceedings: Innovations, Quality & Outcomes. 2019;3(3):358-75.

15.	de Kleijn WP, De Vries J, Lower EE, Elfferich MD, Baughman RP, Drent M. Fatigue in sarcoidosis: a systematic review. Current Opinion in Pulmonary Medicine. 2009;15(5):499-506.

16.	Michielsen HJ, Peros-Golubicic T, Drent M, De Vries J. Relationship between Symptoms and Quality of Life in a Sarcoidosis Population. Respiration. 2007;74(4):401-5.

17.	Bakkers M, Merkies IS, Lauria G, Devigili G, Penza P, Lombardi R, et al. Intraepidermal nerve fiber density and its application in sarcoidosis. Neurology. 2009;73(14):1142-8.

18.	Department of Health and Aged Care. What we’re doing about rare diseases 2022 [updated 29 June 2022. Available from: <https://www.health.gov.au/health-topics/chronic-conditions/what-were-doing-about-chronic-conditions/what-were-doing-about-rare-diseases>.

19.	Iriarte A, Rubio-Rivas M, Villalba N, Corbella X, Mañá J. Clinical features and outcomes of asymptomatic pulmonary sarcoidosis. A comparative cohort study. Respiratory Medicine. 2020;169.

20.	Costabel U, Hunninghake G. ATS/ERS/WASOG statement on sarcoidosis. Sarcoidosis Statement Committee. American Thoracic Society. European Respiratory Society. World Association for Sarcoidosis and Other Granulomatous Disorders. European Respiratory Journal. 1999;14(4):735-7.

21.	Rossides M, Kullberg S, Eklund A, Grunewald J, Arkema EV. Sarcoidosis diagnosis and treatment in Sweden: A register-based assessment of variations by region and calendar period. Respiratory Medicine. 2020;161:105846.

22.	Scott TF, Yandora K, Kunschner LJ, Schramke C. Neurosarcoidosis Mimicry of Multiple Sclerosis: Clinical, Laboratory, and Imaging Characteristics. The Neurologist. 2010;16(6):386-9.

23.	Judson MA, Thompson BW, Rabin DL, Steimel J, Knattereud GL, Lackland DT, et al. The diagnostic pathway to sarcoidosis. Chest. 2003;123(2):406-12.

24.	Veritas Health Innovation. Covidence systematic review software Melbourne, Australia [Available from: Available at www.covidence.org.

25.	Sterne JAC, Sutton AJ, Ioannidis JPA, Terrin N, Jones DR, Lau J, et al. Recommendations for examining and interpreting funnel plot asymmetry in meta-analyses of randomised controlled trials. BMJ. 2011;343:d4002.
